# Recovery and Protective Effect of Direct Transcutaneous Electrical Nerve Stimulation in the Treatment of Acute and Subacute Fibular Tunnel Syndrome

**DOI:** 10.3390/jcm14124247

**Published:** 2025-06-14

**Authors:** Mustafa Al-Zamil, Inessa A. Minenko, Natalia A. Shnayder, Marina M. Petrova, Zarina M. Babochkina, Darya S. Kaskaeva, Vladimir G. Lim, Olga V. Khripunova, Irina P. Shurygina, Natalia P. Garganeeva

**Affiliations:** 1 Department of Physiotherapy, Faculty of Continuing Medical Education, Peoples’ Friendship University of Russia, 117198 Moscow, Russia; 2 Department of Restorative Medicine and Neurorehabilitation, Medical Dental Institute, 127253 Moscow, Russia; kuz-inna@mail.ru (I.A.M.); zzangieva2008@yandex.ru (Z.M.B.); 3 Department of Sports Medicine and Medical Rehabilitation, I.M. Sechenov First Moscow State Medical University, 119991 Moscow, Russia; lim_vg@yahoo.com (V.G.L.); olaw@bk.ru (O.V.K.); 4 Institute of Personalized Psychiatry and Neurology, V.M. Bekhterev National Medical Research Centre for Psychiatry and Neurology, 192019 Saint Petersburg, Russia; 5 Shared Core Facilities “Molecular and Cell Technologies”, Professor V. F. Voino-Yasenetsky Krasnoyarsk State Medical University, 660022 Krasnoyarsk, Russia; stk99@yandex.ru; 6 Department of Outpatient Care and Family Medicine, Professor V. F. Voino-Yasenetsky Krasnoyarsk State Medical University, 660022 Krasnoyarsk, Russia; dashakas.ru@mail.ru; 7 Department of Ophthalmology, Rostov State Medical University, 344022 Rostov, Russia; ir.shur@yandex.ru; 8 Department of General Medical Practice and Outpatient Therapy, Siberian State Medical University, 634050 Tomsk, Russia; garganeeva@gmail.com

**Keywords:** transcutaneous electrical nerve stimulation, TENS, common peroneal nerve, fibular tunnel syndrome, neuropathy, recovery

## Abstract

**Background:** Previous studies have indicated that transcutaneous electrical nerve stimulation (TENS) is highly effective in improving the treatment of neuropathy and achieving maximum recovery in the shortest time. However, its effectiveness in the early stages of the disease has not been studied, and no comparative analysis has been conducted between different modalities of TENS. **Materials and Methods:** This study included 82 patients with acute and subacute fibular tunnel (FT) syndrome lasting no more than 15 days. Patients were randomized into the following four groups depending on the modality of TENS used: sham TENS (20 patients), HF TENS (20 patients), LF TENS (21 patients), and a combined HF/LF TENS group (21 patients). Before treatment, immediately after treatment, and 3 months after the end of treatment patients were examined to determine the severity of hypoesthesia, motor deficit, and gait disturbance. **Results:** The reduction in hypoesthesia averaged after HF TENS, LF TENS, and sham TENS was 50.7% (*p* ≤ 0.01), 37.8 (*p* ≤ 0.01), and 11.4% (*p* > 0.05), respectively. The regression of motor deficit and gate disorders reached 61% after LF TENS (*p* ≤ 0.01), 6% after HF TENS (*p* > 0.05), and 6% (*p* > 0.05) after sham TENS. The combination of HF and LF TENS resulted in a 54.8% (*p* ≤ 0.01) reduction in hypoesthesia and 61.3% (*p* ≤ 0.01) regression of motor deficit, with a superior 30% (*p* ≤ 0.05) improvement in quality of life compared to separate use of HF and LF TENS. **Conclusions:** Early use of TENS in the treatment of FT syndrome turned out to be highly effective compared to sham TENS in reducing hypoesthesia, motor deficit, and gait disturbance. The analgesic effect and sensory recovery were higher after HF TENS. Motor and gait disturbances were reduced only after LF TENS, with evidence of prolonged regenerative and protective effect for at least 3 months after the end of treatment. The combination of HF TENS and LF TENS increases the therapeutic range of TENS with the achievement of the maximum positive effect of HF TENS and LF TENS after treatment and during the long-term period, which leads to a more pronounced improvement in the quality of life of patients with this pathology.

## 1. Introduction

Common peroneal neuropathy is the most commonly diagnosed neuropathy of the lower extremities [1]. Compression of the common peroneal nerve (CPN) most often develops along its passage through the fibular tunnel (FT). The most common names for this neuropathy are peroneal tunnel syndrome and FT syndrome [2]. The relevance of this problem is that common peroneal neuropathy often affects young people of working age, which negatively affects their performance and productivity [3] and has recently become more often diagnosed in children, especially during the coronavirus disease-19 (COVID-19) pandemic due to physical inactivity and prolonged immobilization [4].

Anatomical fibular tunnel is a fibro-osseous channel 3.2 ± 1.0 cm long [5] which is formed by the superficial head of the peroneus longus muscle (arch) and sulcus malleolaris of the fibula and the lower part of the posterior crural intermuscular septum (floor) [6,7]. Through the FT, the CPN passes extremely close to the lateral surface of the fibular neck and in its path comes very close to the sulcus malleolaris and seems to lie on it, which is the most common localization of nerve compression [2].

Compression of the nerve in the FT can be chronic or acute, depending on the duration of compression and development of symptoms. Compression of the CPN usually occurs after mechanical compression of the fibular tunnel due to sports exercises in athletes and dancers [8], wearing tight clothing or tight-fitting legwear [9], prolonged squatting [10], as well as regular crossing of the legs [11]. However, the most common cause of acute nerve compression is nerve compression during deep sleep or long-term bed rest [12].

As a result of compression, the first pathomechanism of tunnel syndromes develops: increasing swelling of the nerve and progression of tunnel stenosis. At this time, patients often experience a feeling of discomfort and numbness in the projection of the FT, the ventrolateral surface of the lower leg and dorsal surface of the foot, which develop quickly within a few hours [12,13]. As the narrowing progresses to absolute stenosis, the nerve is further compressed due to the pressure of the tunnel walls. Subsequently, inflammatory reactions such as localized segmental demyelination, anterograde degeneration (axonotmesis), and endoneural and perineural collagen proliferation accelerate and develop more rapidly impaired axonal transport, ischemic axonopathy, and muscle denervation. Contemporaneously, patients often experience pain in the lower leg and gait disturbances [9,14].

In most cases, the diagnosis of FT syndrome can be suspected based on the characteristic steppage gait and foot drop, which are acutely and rapidly progressive due to weakness of ankle dorsiflexor muscles (m. tibialis anterior, m. extensor digitorum longus, and m. extensor hallucis longus) [15]. Unilateral steppage gait is very sensitive to this disease and can be observed even with moderate dorsiflexion motor deficit due to the inability to fully lift the foot while walking [16]. Altered sensation may be detected in the upper lateral and lower anterolateral surfaces of the leg, as well as in the dorsum of the foot [17].

Other disorders may mimic a common peroneal neuropathy, such as sciatic nerve injury, L5 radiculopathy, distal polyneuropathy, and amyotrophic lateral sclerosis [18]. In this regard, the use of electromyography (EMG) is mandatory and decisive for confirming the diagnosis of FT syndrome. The main EMG criterion of FT syndrome is a decrease in the amplitude of complex muscle action potentials (CMAPs) by more than 20% in response to stimulation of the CPN above the FT and a focal slowing of motor conduction velocity through FT of more than 40 m/s [19]. However, diagnosis of FT syndrome can be challenging in many cases. Misdiagnosis of FT syndrome may result from incorrect assessment of ankle inversion, especially in cases with slight dorsiflexion, as well as inaccurate interpretation of the obtained anamnestic, clinical, and neurophysiological data [20].

Treatment of FT syndrome begins with a conservative approach. However, according to some data, an indication for considering surgery after active conservative treatment is the absence of a positive effect after 2 months or a weak and slow improvement after 4 months [21]. Sensory and motor recovery of an acutely compressed nerve, depending on the degree of damage, can last from 4 weeks to many years. However, nerve recovery becomes significantly slower after 3 months from the onset of the disease.

In many cases, even active adequate pharmacotherapy is ineffective in the treatment of FT syndrome and is ultimately complicated by the gradual development of irreversible nerve axonopathy and atrophic muscle denervation. This outcome can still be avoided by including additional nonpharmacological therapies such as low-level laser therapy [22], extracorporeal shock wave therapy [23], electrical stimulation therapy [24], and magnetic stimulation [25], which are used with high efficiency in the treatment of various forms of neuropathies, achieving the maximum recovery effect in the shortest possible time. Noteworthy, the high therapeutic effect of transcutaneous electrical nerve stimulation (TENS) has been reported in many studies [26,27,28]. In addition to the segmental and suprasegmental analgesic effect, numerous experimental studies have demonstrated the local anti-inflammatory and regenerative effect of TENS. In the area of TENS application, an increase in arterial and microcirculatory blood flow [29,30], venous hemodynamic response [31], a decrease in the level of pro-inflammatory cytokines [32], as well as the secretion and accumulation of growth factors [33] were revealed. Ultimately, these mechanisms lead to an increase in the number and diameter of axons, which is associated with remyelination and axonal recovery [34].

To the best of our knowledge, there are no studies examining the efficiency of TENS in the treatment of FT syndrome in the early stages of the disease. In addition, to date there are no studies devoted to a comparative analysis of various modalities of TENS in the treatment of this pathology. In response to this challenge, the goal of this research was to investigate the efficacy of different modalities of direct TENS in reducing sensory and motor deficits in the treatment of FT syndrome within two weeks of the onset of neuropathy.

## 2. Materials and Methods

### 2.1. Study Design and Population

This interventional single-blind, four-armed randomized controlled trial followed 82 patients with FT syndrome.

#### 2.1.1. Selection Criteria

The inclusion criteria were as follows:

1. European patients;
2. Adult men and women from 21 to 60 years old;
3. Duration of FT syndrome is less than 15 days;
4. Unilateral lesion;
5. Conduction block of common peroneal nerve at the FT level > 30% and/or decreased in motor common peroneal nerve conduction velocity < 40 m/s;
6. Ankle dorsiflexion weakness is maximum 4/5 points, patients with paralysis are not included in this study;
7. Voluntary consent to participate in this study.

The exclusion criteria were as follows [26]:

1. Epilepsy;
2. Cognitive and mental disorders;
3. Distal polyneuropathy or L5 radiculopathy;
4. Brain and spinal cord disorders;
5. Cardiac arrhythmias and cardiac pacemaker;
6. Botulinum toxin injections to any muscle of leg in the previous 3 months;
7. Vascular atherosclerosis and venous thrombosis of the lower extremities;
8. Diabetes mellitus;
9. Undergoing another physiotherapy treatment or acupuncture;
10. Pregnancy.

#### 2.1.2. Ethical Statement and Registration Documents

After explaining the purpose of the study and the safety of pharmacotherapy and TENS, all patients signed voluntary informed agreement for participation.

The study was approved by the ethical commission of the Russian Medical Dental Institute (protocol No. 28, 11 November 2022). The research was registered with the Higher Attestation Commission of the Russian Federation (ID: 0120.0807480) and in the international ISRCTN registry (ISRCTN47534508).

The recommendations of the 1984 Helsinki Declaration and subsequent amendments were strictly observed. All patients gave written consent for publication after reading the manuscript in full. No remuneration was paid to participants or investigators in this study. The study is a part of the scientific program of the Department of Restorative Medicine and Neurorehabilitation of the Russian Medical Dental Institute.

#### 2.1.3. Patient Randomization into Groups

A total of 324 subjects (F: 170, M: 154) passed the eligibility assessment. Of these, 232 were excluded. A total of 209 patients did not meet inclusion criteria, and 23 patients declined to participate.

**Figure 1:**
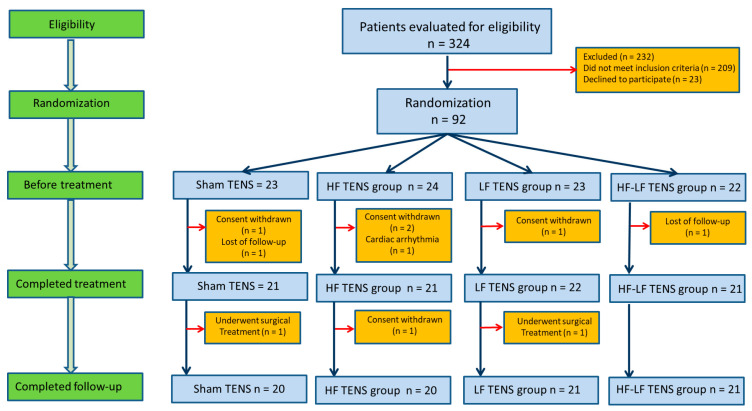
Study population selection diagram. Note: TENS—transcutaneous electrical nerve stimulation; HF—high frequency; LF—low frequency; HF-LF TENS—combination of HF TENS and LF TENS.

As a result, only 92 patients (F: 50, M: 42) were randomized into four groups depending on the modality of TENS used. A total of 23 patients (F: 12, M: 11) underwent sham TENS (sham TENS group), 24 patients (F: 14, M: 10) underwent high-frequency low-amplitude TENS (HF TENS group), 23 patients (F: 12, M: 11) underwent low-frequency high-amplitude TENS (LF TENS group), and 22 patients (F: 12, M: 10) underwent a combination of HF TENS and LF TENS (HF-LF TENS group). However, 7 patients were excluded from the study due to loss during follow-up (*n* = 2), withdrawal of consent (*n* = 4), and development of cardiac arrhythmia in 1 patient. Correspondingly, 21 patients completed treatment in the sham TENS group, 21 patients in the HF TENS group, 22 patients in the LF TENS group, and 21 patients in the HF-LF TENS group. After completion of the course of treatment, patients were observed for 3 months. Follow-up was completed in 20 patients in the sham group, 20 patients in the HF TENS group, 21 patients in the LF TENS group, and 21 patients in the HF-LF TENS group. Two patients underwent surgery, and one patient withdrew consent (Figure 1). To simplify the comparative analysis, we used the term “active TENS group” for HF TENS, LF TENS, and HF-LF TENS.

#### 2.1.4. Demographic and Clinical Characteristics

Demographic and clinical aspects of included patients are summarized in Table 1. The patients’ ages ranged from 21 to 59 years, with an average of 40.5 ± 1.41 years. In all study groups the ratio of female to male was 1:1. FT syndrome duration lasted from 3 to 14 days and averaged 8.13 ± 0.4 days. Ankle dorsiflexion weakness varied from 1 to 4 points, with an average value of 3.14 ± 0.12 points. Steppage gait disorders varied from 1 to 3 points and averaged 2.88 ± 0.04 points. The severity of hypoesthesia in the anterolateral surface of the lower leg was assessed using VAS from 4 to 10 points; the average result was 6.21 ± 0.16 points. No significant differences were registered in gender, age, disease duration, ankle dorsiflexion weakness, steppage gait disorder, and severity of hypoesthesia between the groups.

### 2.2. Sample Size Calculation

A previous study [26] showed that in patients with persistent sensory deficit after carpal tunnel release TENS resulted in improvement in sensory function from a baseline score of 3.4 ± 0.58 after HF-TENS to 26.5% and after LF-TENS to 14.7%, with a mean improvement of 20.6% in the TENS groups and no improvement in the control group. According to these results, the sample size of our study was calculated [35]. As a result, a sample size calculation with power value = 95%, probability of type I error = 0.01, and expected significance level = 0.05 showed that we needed 18 patients or more.

### 2.3. Assessment and Outcome Measures

Neurological examination to assess primary and secondary outcome was performed before initiation of TENS therapy, after TENS therapy, and 3 months after completion of TENS therapy.

#### 2.3.1. Primary Outcome Measure

##### Hypoesthesia

Tactile hypoesthesia on the affected surface of the lower leg and foot was studied using the Semmes–Weinstein monofilament. The severity of tactile hypoesthesia was determined by VAS relative to tactile sensation on the unaffected leg.

##### Motor Deficit

The weakness of ankle dorsiflexion was assessed on the following 5-point scale:

- 5 points: no visible muscle activation.
- 4 points: muscle activation with no or trace movement.
- 3 points: muscle activation but not against gravity.
- 2 points: muscle activation against gravity but not resistance.
- 1 points: muscle activation against some resistance results in full ankle dorsiflexion.
- 0 points: muscle activation against full resistance results in full ankle dorsiflexion.

##### Gait Disorder

The degree of steppage gait disturbance was determined on a 4-point scale depending on the degree to which the heel strikes the ground before the forefoot. Steppage gait is a gait disorder characterized by the inability to lift the foot while walking due to weakness of ankle dorsiflexor muscles [16]. As a result, in severe cases, the forefoot touches the ground first, followed by the heel. Gait disturbance assessment was performed as follows:

- Point 0 corresponds to the absence of gait disturbances. When walking, the angle of contact between the heel and the ground is very noticeable.
- Point 1 corresponds to the moderate gait disturbance in which the heel contacts the ground for a very short period of time before the forefoot contacts the ground.
- Point 2 corresponds to the severe gait disturbance in which the entire foot landing on the ground with a characteristic slap.
- Point 3 corresponds to the maximum gait disturbance, in which the forefoot touches the ground before the heel. To avoid falling due to the inability to lift the toe off the ground and the possible impact of the edge of the forefoot on the floor when stepping with the affected leg, the patient has to lift the foot more than its length, performing a characteristic maneuver resembling a rooster’s gait.

#### 2.3.2. Secondary Outcome Measure

##### Pain Syndrome

Pain was assessed at rest by 10-point visual analog scale (VAS) and by palpation or tapping of the CPN around the fibular head (Tinel’s sign).

##### Paraesthesia

Patients determined the severity of paraesthesia symptoms by VAS. Tingling, numbness, burning, and electric shock-like brief painful sensation in the projection of the fibular head, the ventrolateral surface of the lower leg, and the dorsal side of the foot were measured on a 10-point scale at rest and after walking a distance of 20 m.

##### Global Quality of Life Scale

Similarly to a visual analog scale, the scale has 100 percentage points representing quality of life from 0 to 100, with 0 points indicating “No quality of life” and 100 points indicating “Perfect quality of life” [36]. Unlike other questionnaires, there is no multidimensional or multidomain approach and the patient focuses on the problem that directly reduces quality of life. In addition, conducting this survey does not require computer calculations and does not take much time.

### 2.4. Electromyograohy Examination

Neurophysiological parameters of CPN were studied by EMG examination using evoked and needle techniques. The motor conduction velocity of the CPN was measured bilaterally at the fibular tunnel, lower leg, and foot. Distal latency of CMAP was registered using stimulation at the ankle joint. CMAP amplitude was measured in response to stimulation of the nerve above and below the FT and at the ankle joint. The EMG recordings were made from the extensor digitorum brevis muscle. Conduction study of the superficial peroneal nerve was provided antidromically. Needle EMGs of the tibialis anterior muscle, peroneus longus, vastus lateralis, gastrocnemius (medial head), tensor fascia latae, and biceps femoris (short head) were used to determine active denervation potentials at rest, as well as the recruitment amplitude and duration of the recorded motor potential units (MPUs) at the moderate to maximum muscle contraction. EMG monitoring was performed before the start of TENS therapy and 3 months after the completion of TENS therapy. The EMG device “Neuroexpeditor” from the MBN company was used (registration number: FSR 2010/07889).

### 2.5. Ultrasonography of Peroneal Nerve

In our study, all patients underwent an ultrasound diagnostic of CPN before treatment an at the end of follow–up. The studies were carried out on a Samsung Medison UGEO H60 ultrasound machine, registration number P3H 2013/691, dated 10 July 2017.

### 2.6. Pharmacotherapy

Simultaneously with the start of the TENS course, all patients received pharmacotherapy with the following drugs orally: ipidacrine 60 mg per day for 1 month, alpha-lipoic acid 600 mcg per day for 2 months, pentoxifylline 300 mcg per day for 1 month, and cyanocobalamin 1000 mcg per day intramuscularly for 10 days. No allergic reactions or side effects to the drug therapy used have been registered.

### 2.7. Direct Transcutaneous Electrical Nerve Stimulation

In each group, TENS was performed using different techniques, as shown in Table 2. TENS was delivered using a BTL-4000 smart device (BTL Industries Ltd., Stoke-on-Trent, UK) with registration number RAN 2020/12648, dated 24 November 2020.

#### 2.7.1. Protocol of Sham TENS

Stable and labile TENS were performed as indicated in Table 2. We used 1 Hz and 50 μs monophasic rectangular current with an amplitude of 1 mA, exceeding the sensation threshold. Stimulation was administered for 30 min, including 15 min for stable TENS and 15 min for labile TENS. The procedures were performed 20 times every other day.

#### 2.7.2. Protocol of HF TENS

Stable and labile TENS was performed as indicated in Table 2. We used 50 Hz and 50 μs monophasic rectangular current with an amplitude of 1 mA, exceeding the sensation threshold. Stimulation was administered for 30 min, including 15 min for stable TENS and 15 min for labile TENS. The procedures were performed 20 times every other day.

#### 2.7.3. Protocol of LF TENS

**Figure 2:**
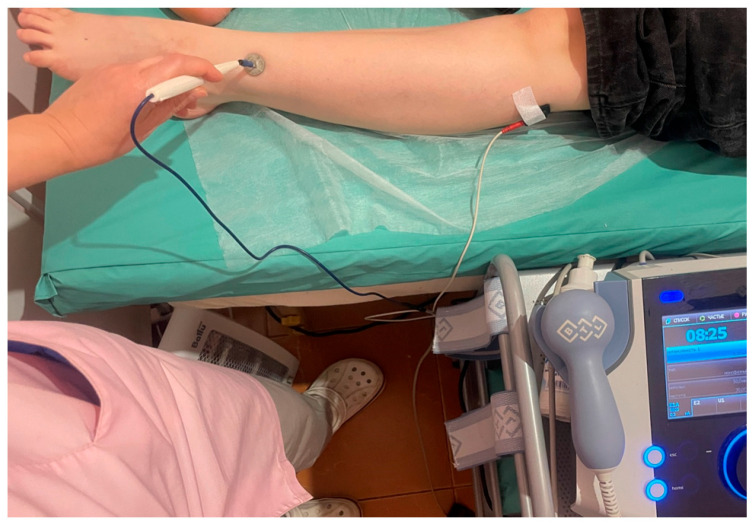
Technique of labile transcutaneous electrical nerve stimulation of the left peroneal nerve.

Stable and labile TENS was performed as indicated in Table 2. We used 1 Hz and 200 μs monophasic rectangular current with an amplitude of 1 mA, exceeding the comfortable painless motor response. Stimulation was administered for 30 min, including 15 min for stable TENS and 15 min for labile TENS. The procedures were performed 20 times every other day (Figure 2).

#### 2.7.4. Protocol of HF-LF TENS

A combined application of HF and LF TENS was used, observing the above-mentioned characteristics for each modality, with the exception of the time characteristics, which were 15 min for HF TENS (7.5 min for stable TENS and 7.5 min for labile TENS) and 15 min for LF TENS (7.5 min for stable TENS and 7.5 min for labile TENS). The procedures were performed 20 times every other day.

### 2.8. Statistical Analysis

The interventional factorial four-arm single-blind fixed block randomization method was used in SPSS software for Windows (version 20). Blinding assessment was not conducted during the trial study. The primary endpoints of our study are hypoesthesia, motor deficit, and gait disorders, which were indicated in the purpose and in the Materials and Methods Section. The secondary endpoints are pain, paraesthesia, and quality of life. To address different baseline values between groups when statistically analyzing the secondary outcome measures, we used data transformations, normalization, and outlier capping. These methods aim to make the data more comparable and reduce the impact of initial differences, allowing for a more accurate analysis of the effect of an intervention or other factors. The results of EMG examination are exploratory.

For comparative analysis between groups, mean values (M), standard deviation (SD), and standard error of the mean (SEM) were calculated. To assess whether a dataset is normally distributed the Shapiro–Wilk test was performed. To assess whether the variances of two or more groups are equal Levene’s test was applied. To reduce the chances of type I errors, the Bonferroni correction test was used. Analysis of variance (ANOVA) was used to compare the mean values of two or more groups on one of the following single dependent variables: hypoesthesia, motor deficit, gait disturbance, paresthesia, pain, and quality of life. The method was mainly used to detect if there are statistically significant differences between the group means before treatment.

To determine the significant difference and the relationship between the means of two groups, a *t*-test was performed. For example, between HF TENS and LF TENS, between LF TENS and sham TENS, etc. A difference with *p* ≤ 0.05 was considered statistically significant.

Multivariate analysis of variance MANOVA was used to analyze significant differences between multiple outcomes in four studied groups. Using this method, we attempted to determine comparisons between groups on several clinical manifestations such as pain, neurological deficit, and quality of life. However, the results were not significant. Non-parametric tests were not used in our study.

## 3. Results

### 3.1. Primary Clinical Outcomes

#### 3.1.1. Dynamics of Hypoesthesia

**Figure 3:**
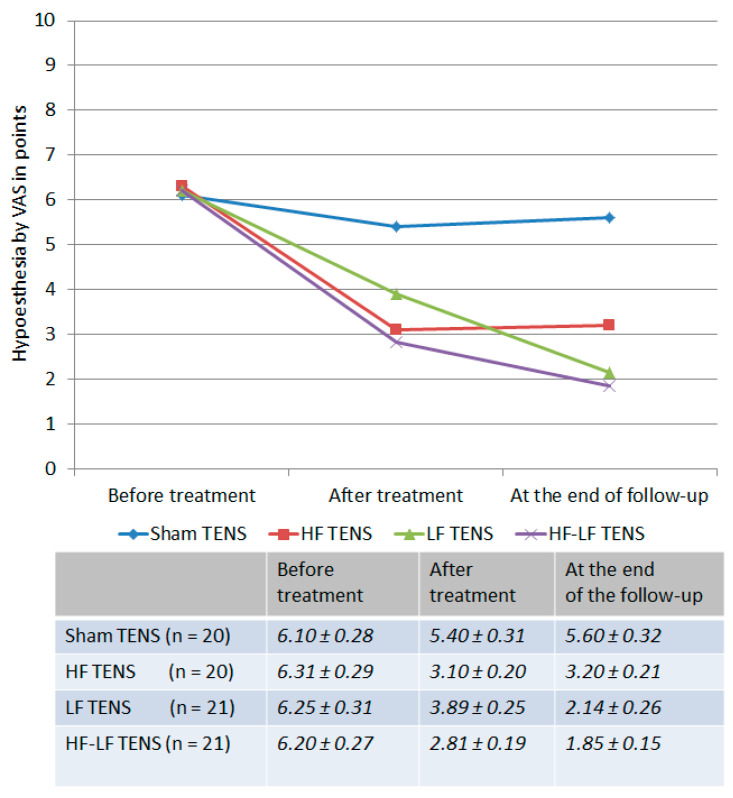
Dynamics of hypoesthesia in all study groups immediately after completion of the course of transcutaneous electrical nerve stimulation and at the end of the 3 month follow-up. Note: TENS—transcutaneous electrical nerve stimulation; HF—high frequency; LF—low frequency; HF-LF TENS—combination of HF TENS and LF TENS.

Before treatment: The loss of tactile sensation in the ventrolateral surface of the affected lower leg compared with the unaffected side averaged 6.22 ± 0.18 points (Figure 3). No remarkable differences were found between study groups.

After treatment: In the active TENS groups a 47.8% (t = 9.01, *p* ≤ 0.01) reduction in hypoesthesia was recorded, whereas this did not occur in the sham TENS group.

Post-treatment follow-up: Tactile sensation improved in the LF TENS and HF-LF TENS groups compared to the post-treatment level by 45% (t = 4.85, *p* ≤ 0.01) and 34.2% (t = 3.92, *p* ≤ 0.01), respectively, and did not change markedly in the HF TENS group. As a result, hypoesthesia was less than the pre-treatment level in the HF TENS group by 49.3% (t = 8.69, *p* ≤ 0.01), in the LF TENS group by 65.8% (t = 10.1, *p* ≤ 0.01), and in the HF-LF TENS group by 70.2% (t = 14.1, *p* ≤ 0.01).

Comparative analysis between groups

The use of active TENS has a significant advantage over sham TENS by 39.5% (t = 4.95, *p* ≤ 0.01) immediately after treatment and by 43% (t = 4.95, *p* ≤ 0.01) at the end of the follow-up.

The severity of hypoesthesia after treatment was lower by 20.3% (t = 2.47, *p* ≤ 0.05) after HF TENS compared with LF TENS, while the regression of hypoesthesia at the end of the follow-up was higher after LF TENS by 33.1% (t = 3.17, *p* ≤ 0.05). The maximum reduction in hypoesthesia obtained in the HF-TENS group immediately after treatment and in the LF-TENS group 3 months after the completion of TENS therapy did not differ remarkably from the treatment results in the HF-LF-TENS group.

#### 3.1.2. Changes in Ankle Dorsiflexion Strength

**Figure 4:**
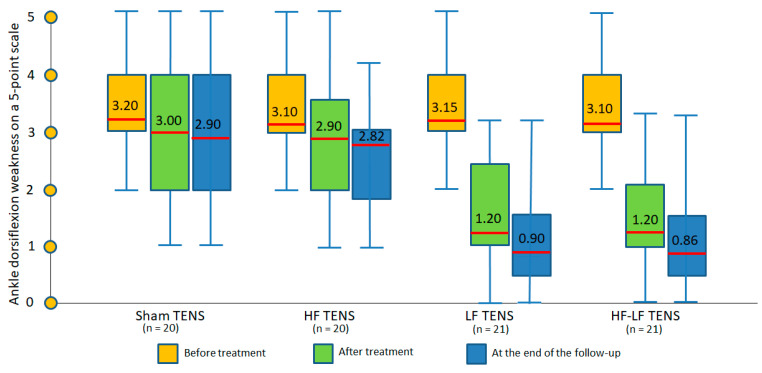
Dynamics of ankle dorsiflexion weakness in all study groups immediately after completion of the course of transcutaneous electrical nerve stimulation and at the end of the 3 month follow-up. Note: TENS—transcutaneous electrical nerve stimulation; HF—high frequency; LF—low frequency; HF-LF TENS—combination of HF TENS and LF TENS.

Before treatment: Ankle dorsiflexion weakness ranged from 1 to 4 points and averaged 3.14 ± 0.14 points. The severity of motor impairment was largely the same in different groups (Figure 4).

After treatment: The regression in motor deficit was recorded only in the LF TENS and HF-LF TENS groups, where it fell by 61.9% (8.322, *p* ≤ 0.01) and 61.3% (8.45, *p* ≤ 0.01), respectively. No significant changes were recorded in the sham TENS and HF TENS groups.

Post-treatment follow-up: No significant negative dynamics were observed in any of the study groups. Compared with pre-treatment levels, weakness was reduced to 71.4% (t = 9.86, *p* ≤ 0.01) in the LF TENS groups and to 72.3% in the HF-LF TENS group (t = 9.49, *p* ≤ 0.01). No improvement in motor function was recorded in either the sham TENS or HF TENS groups.

Comparative analysis between groups

Regression of motor deficit was exclusively registered after the use of effective TENS, but not after the use of sham TENS.

LF TENS but not HF TENS resulted in a significant reduction in ankle dorsiflexion weakness. However, the combination with HF TENS did not lead to an enhancement in the recovery effect of LF TENS either after the treatment or 3 months after the completion of TENS therapy.

#### 3.1.3. Gait Disorders Assessment

**Figure 5:**
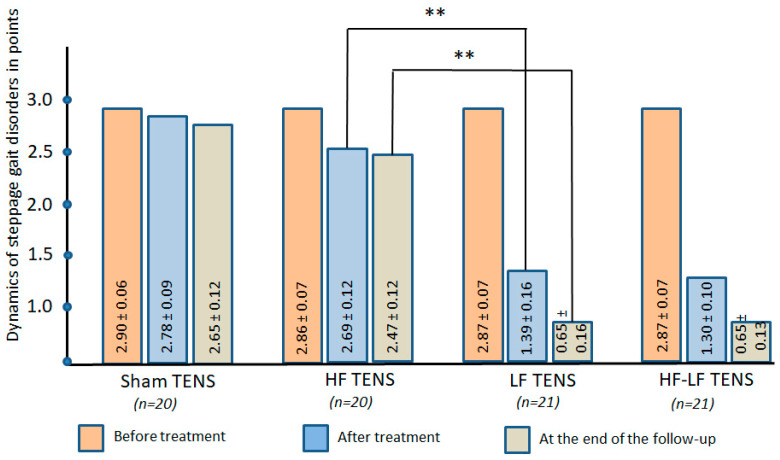
Dynamics of steppage gait disorders in all study groups immediately after completion of the course of transcutaneous electrical nerve stimulation and at the end of the 3 month follow-up. Note: TENS—transcutaneous electrical nerve stimulation; HF—high frequency; LF—low frequency; HF-LF TENS—combination of HF TENS and LF TENS; **—*p* ≤ 0.01.

Before treatment: In 100% of the patients, a steppage gait disturbances was diagnosed, which ranged from 1 to 3 points and averaged 2.88 ± 0.04 points. No notable differences were recorded across the study groups (Figure 5).

After treatment: Similar to the results obtained in the study of the motor sphere, improvement in gait was noted appreciably only in the HF-LF TENS and LF-TENS groups, where it improved by 51.6 (t = 8.47, *p* ≤ 0.01) and 54.7% (t = 7.75, *p* ≤ 0.01), and improvement was not detected in the sham TENS and HF-TENS groups.

Post-treatment follow-up: Gait continued to improve even after treatment in the LF TENS and HF-LF TENS groups was discontinued, and it was enhanced by an average of 52.1% (t = 3.00, *p* ≤ 0.01) over the post-treatment level. Compared with pre-treatment levels, gait improvement reached 77.3% (t = 12.7, *p* ≤ 0.01) in the LF TENS group and 77.3% (t = 15.3, *p* ≤ 0.01) in the HF-LF TENS group and was negligible in the sham TENS and HF TENS groups.

Comparative analysis between groups

In contrast to the noteworthy results of active TENS, no positive gait dynamics were observed after using sham TENS.

Comparative analysis of HF TENS and LF TENS showed that only LF TENS resulted in improvement in gait disturbances without significant differences from the results of HF-LF TENS in all observation periods.

### 3.2. Secondary Clinical Outcomes

#### 3.2.1. Pain Syndrome at Rest

**Figure 6:**
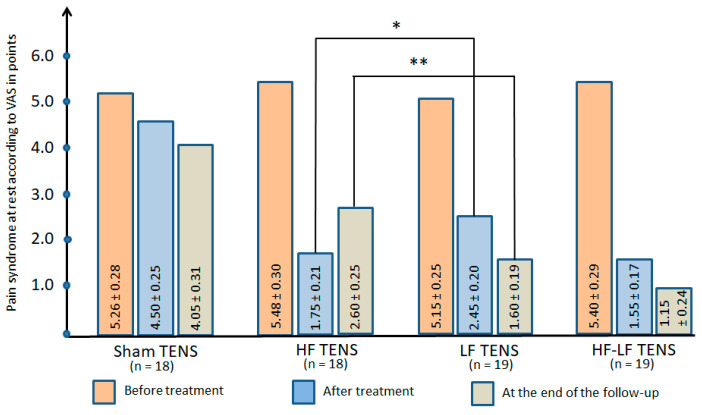
Dynamics of pain syndrome at rest in all study groups immediately after completion of the course of transcutaneous electrical nerve stimulation and at the end of the 3 month follow-up. Note: TENS—transcutaneous electrical nerve stimulation; HF—high frequency; LF—low frequency; HF-LF TENS—combination of HF TENS and LF TENS; *—*p* ≤ 0.05; **—*p* ≤ 0.01.

Before treatment: At rest, pain in the projection of the fibular tunnel and along the anterolateral surface of the lower leg in all groups varied from 3 to 10 points and averaged 5.32 ± 0.17 points. No significant differences were found between the study groups (Figure 6).

After treatment: A significant analgesic effect was found in all groups and amounted to 14.4% (t = 2.0247, *p* < 0.05) in the sham TENS group, 68.0% (t = 10.1858, *p* ≤ 0.01) in the HF TENS group, 52.4% (t = 8.4334, *p* ≤ 0.01) in the LF TENS group, and 71.3% (t = 11.4531, *p* ≤ 0.01) in the HF-LF TENS group.

Post-treatment follow-up: Compared with post-treatment levels, a reliable increase in the analgesic effect in the LF TENS group by 34.7% (t = 3.0812, *p* ≤ 0.05) was noted, with a significant decrease in the analgesic effect in the HF TENS group by 48,6% (t = 2.60, *p* ≤ 0.05), with no reliable dynamic changes in the analgesic effect after sham TENS and HF-LF TENS. However, in all of the study groups, pain syndrome was lower than the pre-treatment levels, reducing by 23% (t = 2.89, *p* ≤ 0.01) in the sham TENS group, by 52.6% (t = 7.37, *p* ≤ 0.01) in the HF TENS group, by 68.9% (t = 11.3, *p* ≤ 0.01) in the LF TENS group, and by 78.7% (t = 11.3, *p* ≤ 0.01) in the HF-LF TENS group.

##### Comparative Analysis Between Groups

After active TENS a 57.4% (t = 5.34, *p* ≤ 0.01) greater analgesic effect was observed compared to sham TENS and amounted to 56.0% (t = 5.47, *p* ≤ 0.01) 3 months after completion of TENS therapy.

HF TENS was 28.6% (t = 2.41, *p* ≤ 0.05) more effective than LF TENS in relieving pain after treatment. However, the inconsistency of the analgesic effect of HF TENS resulted in the superiority of LF TENS by 38.5% (t = 3.18, *p* ≤ 0.01) at the end of the follow-up. These results did not significantly differ from the treatment results from HF-LF TENS. However, at the end of the follow-up, the analgesic effect after HF-LF TENS was 55.8% (t = 4.18, *p* ≤ 0.01) stronger.

#### 3.2.2. Dynamics of Tinel’s Sig

**Figure 7:**
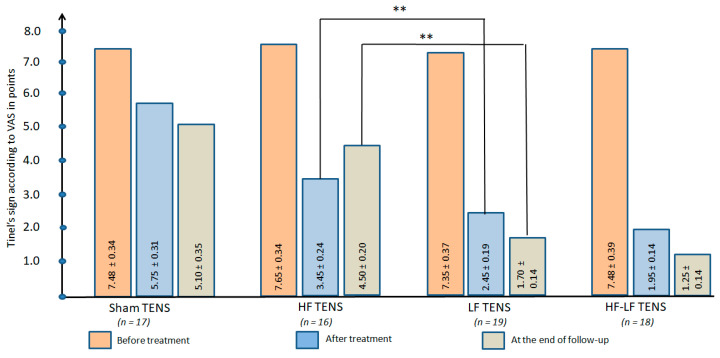
Dynamics of Tinel’s sign in all study groups immediately after completion of the course of transcutaneous electrical nerve stimulation and at the end of the 3 month follow-up. Note: TENS—transcutaneous electrical nerve stimulation; HF—high frequency; LF—low frequency; HF-LF TENS—combination of HF TENS and LF TENS. **—*p* ≤ 0.01.

Before treatment: Pain syndrome or a sensation of tingling and needles in response to light tapping over the CPN averaged 7.49 ± 0.2 points in all groups (Figure 7).

After treatment: Tinel’s sign regression was observed in all groups and was 23.1% (t = 3.7600, *p* ≤ 0.01) after sham TENS, 54.9% (t = 11.7808, *p* ≤ 0.01) after HF TENS, 66.7% (t = 10.1, *p* ≤ 0.01), and 73.9% (t = 13.3, *p* ≤ 0.01) after HF-LF TENS.

Post-treatment follow-up: Despite the cessation of treatment, Tinel’s symptoms continued to decrease in the LF TENS and HF-LF TENS groups by an average of 33.2% (t = 3.1779, *p* ≤ 0.01). In contrast, in the HF TENS group a negative trend of 30% was recorded (t = 3.20, *p* ≤ 0.01). No significant dynamics was noted in the sham TENS group. Overall, the results obtained were lower than pre-treatment levels by 31.8% (t = 4.8775, *p* ≤ 0.01) in the sham TENS group, by 41.2% (t = 13.2, *p* ≤ 0.01) in the HF TENS group, by 76.9% (t = 14.3, *p* ≤ 0.01) in the LF TENS group, and by 83.3% (t = 15.0, *p* ≤ 0.01) in the HF-LF TENS group.

##### Comparative Analysis Between Groups

The reduction in Tinel’s sign was more pronounced in the effective TENS groups compared to the sham TENS group by 54.5% (t = 9.10, *p* ≤ 0.01) immediately after treatment and by 51.3% (t = 7.50, *p* ≤ 0.01) by the end of follow-up.

At the end of treatment, HF-TENS had a 40.8% (t = 3.27, *p* ≤ 0.01) greater analgesic effect than LF-TENS, with a gradual decrease in this effect during the follow-up, resulting in a 62.2% (t = 3.07, *p* ≤ 0.01) advantage for LF-TENS. However, the combination of LF TENS with HF TENS increased the analgesic effect by 43.4% (t = 5.39, *p* ≤ 0.01) immediately after treatment and by 26.7% (t = 2.27, *p* ≤ 0.05) at the end of the follow-up.

#### 3.2.3. Paraesthesia at Rest

**Figure 8:**
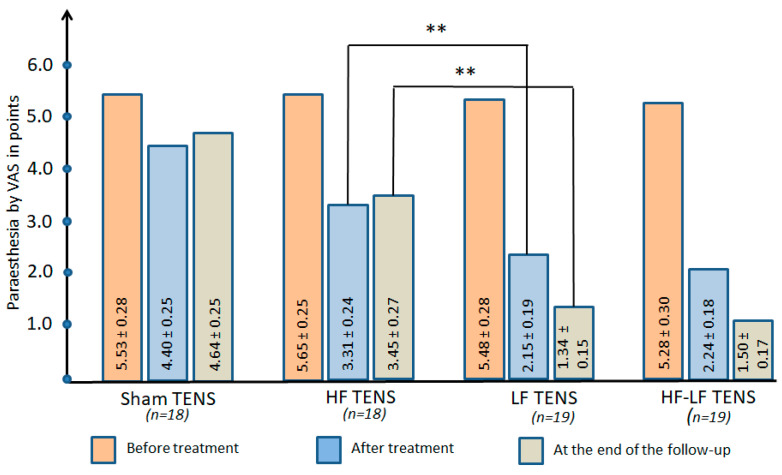
The dynamics of paraesthesia at rest in all of the study groups immediately after completion of the course of transcutaneous electrical nerve stimulation and at the end of the 3 month follow-up. Note: TENS—transcutaneous electrical nerve stimulation; HF—high frequency; LF—low frequency; HF-LF TENS—combination of HF TENS and LF TENS; **—*p* ≤ 0.01.

Before treatment: The mean paraesthesia score (tingling, numbness, burning, and electric shock sensation) in all groups was 5.49 ± 0.17 points, with no significant differences between groups (Figure 8).

After treatment: Paraesthesia decreased most often in the effective TENS groups by an average of 53.3% (t = 7.32, *p* ≤ 0.01) and decreased after the sham TENS group by only 20.4% (t = 3.0, *p* ≤ 0.01).

Post-treatment follow-up: The reduction in paraesthesia symptoms continued after the finish of TENS treatment in only the LF TENS and HF-LF TENS groups, recording rates of 37.7% (t = 3.35, *p* ≤ 0.01) and 33.0% (t = 2.99, *p* ≤ 0.01), respectively. However, compared with pre-treatment values, paraesthesia was lower after sham TENS by 16.1% (t = 2.52, *p* ≤ 0.05) and after active TENS by 62% (t = 11.2, *p* ≤ 0.01).

##### Comparative Analysis Between Groups

Paresthesia at rest was reduced by 41.7% (t = 6.54, *p* ≤ 0.01) immediately after active TENS and by 54.8% (t = 7.14, *p* ≤ 0.01) 3 months after the completion of TENS therapy when compared to after sham TENS.

LF TENS produced a greater reduction in paresthesia compared to HF TENS immediately after treatment and 3 months after the completion of TENS therapy, with the results showing its reduction was greater by 35.0% (t = 3.79, *p* ≤ 0.01) and 61.2% (t = 6.83, *p* ≤ 0.01), respectively. However, these results were not significantly different from the results of HF-LF TENS application either after treatment or at the end of the follow-up.

#### 3.2.4. Dynamics of Paraesthesia After 20 m of Walking

**Figure 9:**
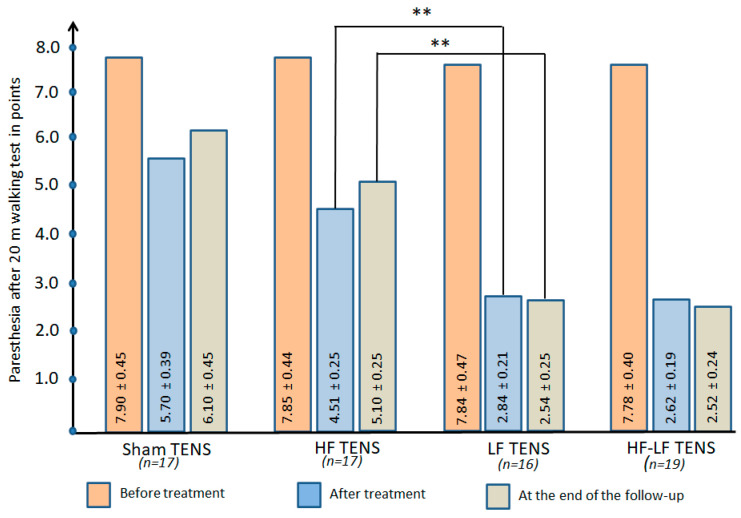
The dynamics of paresthesia after 20 m of walking in all of the study groups immediately after completion of the course of transcutaneous electrical nerve stimulation and at the end of the 3 month follow-up. Note: TENS—transcutaneous electrical nerve stimulation; HF—high frequency; LF—low frequency; HF-LF TENS—combination of HF TENS and LF TENS; **—*p* ≤ 0.01.

Before treatment: Paraesthesia after 20 m of walking increased by 43% (t = 5.32, *p* ≤ 0.01) compared to values at rest and averaged 7.84 ± 0.21 points. No significant differences in paraesthesia severity were found across the four groups (Figure 9).

After treatment: Paresthesia was significantly reduced in all groups, most with LF TENS where it was reduced by 65% (t = 10.2, *p* < 0.01), to a lesser extent with HF TENS where it was reduced by 42.5% (t = 6.62, *p* ≤ 0.01), and the least after sham TENS where it was reduced by 27.8% (t = 3.69, *p* ≤ 0.01).

Post-treatment follow-up: The treatment results had a prolonged effect in all of the groups, especially after LF TENS and HF-LF TENS, which was reflected in a decrease in paraesthesia compared to pre-treatment levels of approximately 67.6% (t = 9.95, *p* ≤ 0.01). However, the level of paresthesia in the sham TENS and HF TENS groups was significantly lower than baseline by 35.0 (t = 5.43, *p* ≤ 0.01) and 22.8% (t = 2.82, *p* ≤ 0.01), respectively.

##### Comparative Analysis Between Groups

Paraesthesia after 20 m of walking decreased by 41.7% (t = 5.51, *p* ≤ 0.01) after undergoing active TENS compared to sham TENS, with a 44.5% (t = 4.48, *p* ≤ 0.01) advantage at the end of the follow-up.

LF-TENS turned out to be more effective than HF-TENS in preventing the development of paraesthesia by 37% (t = 5.11, *p* ≤ 0.01) after treatment and by 50.2% in the long-term period (t = 7.24, *p* ≤ 0.01). Marginal differences were found between the LF TENS and HF-LF TENS groups after treatment and 3 months after the completion of TENS therapy.

#### 3.2.5. Quality of Life

**Figure 10:**
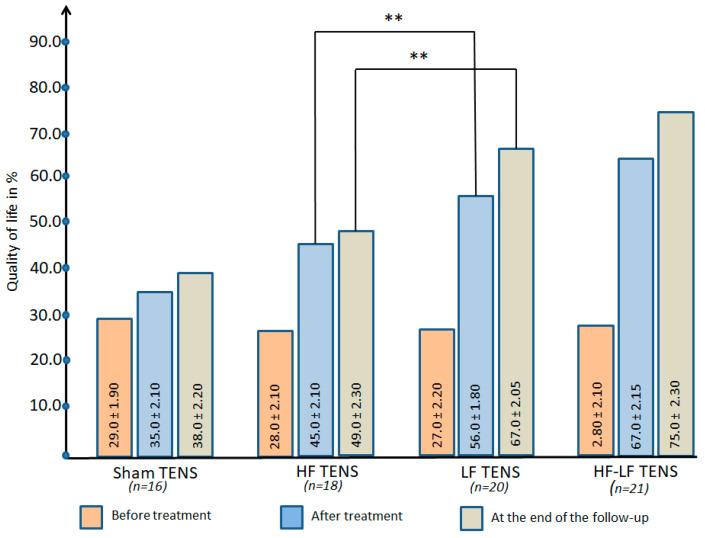
The dynamics of global quality of life in all study groups immediately after completion of the course of transcutaneous electrical nerve stimulation and at the end of the 3 month follow-up. Note: TENS—transcutaneous electrical nerve stimulation; HF—high frequency; LF—low frequency; HF-LF TENS—combination of HF TENS and LF TENS; **—*p* ≤ 0.01.

Before treatment: The quality of life in all of the study groups was low and was at almost the same level, on average 28.0 ± 1.6% (Figure 10).

After treatment: The improvement in quality of life in the active TENS groups averaged 102% (t = 10.2, *p* < 0.01) and did not exceed 20% (t = 2.11, *p* < 0.05) in the sham TENS group.

Post-treatment follow-up: Quality of life in the sham TENS and HF TENS groups was maintained over the 3 month observation period without negative dynamics. At the same time, in the LF TENS and HF-LF TENS groups a continuing improvement in quality of life was noted, which led to an improvement in quality of life of 1.48 and 1.68 times in these groups compared to the pre-treatment level.

##### Comparative Analysis Between Groups

Improvement in quality of life after active TENS was superior to sham TENS by 60.0% (t = 6.11, *p* ≤ 0.01) immediately after treatment and by 67.0% by the end of the follow-up (t = 5.47, *p* ≤ 0.01).

Immediately after treatment and 3 months after completion of TENS therapy, quality of life was higher in the LF TENS group compared to the HF TENS group by 24.4 (t = 4.20, *p* ≤ 0.01) and 36.7% (t = 5.90, *p* ≤ 0.01), respectively. Nevertheless, compared with the HF-LF TENS group, post-treatment and long-term LF TENS levels were 16.4% (t = 6.50, *p* ≤ 0.01) and 11% (t = 2.63 *p* ≤ 0.05) lower, respectively.

### 3.3. Ultrasound Examination of CPN

The CPN was scanned in the popliteal fossa and FT, starting from the bifurcation point of the sciatic nerve. Entrapment of the nerve by myofascial herniation, orthopedic hardware, ganglion cysts, lipomas, thickened fascia, soft tissue tumors, bone tumors, and scar tissue were excluded. Ischemic changes including loss of normal fascicles and epineurium damage, nerve echogenicity, ratio of cross-sectional area of nerve, and internal color Doppler imaging have also been analyzed. Most patients showed signs of acute edema and focal inflammatory process of varying severity. However, these data are not comparable and cannot be used for comparative analysis of treatment results between groups.

### 3.4. Electromyography Findings

Pre-treatment electromyography data varied between patients. However, the main criteria for CPN damage in the fibular tunnel were a conduction block greater than 30% and/or a decrease in the impulse conduction velocity along the motor fibers of the nerve at this level. It is noteworthy that the severity of these abnormalities correlates with the duration and degree of compression of the nerve, which can change over time after the onset of the disease. Consequently, it was difficult to establish an initial point of comparison for each patient. Moreover, it is important to note that the amplitude of CMAP in response to the distal stimulation at the end of treatment in most patients is reduced compared to pre-treatment values, despite positive clinical results. Thus, using CMAP to determine the effectiveness of treatment for each patient is impractical and, in many cases, may be misleading. However, comparative analysis of CMAP amplitude between both sides provides a more objective evaluation of the damaged motor unit of the affected CPN, given that CMAP amplitude is directly proportional to the number of motor units. Accordingly, we decided to determine the CMAP amplitude in response to the stimulation of the distal deep peroneal nerves on both legs 3 months after the completion of TENS therapy. The relative decrease in CMAP amplitude on the affected side was subsequently calculated. The obtained results were used as an objective neurophysiological indicator of the treatment effectiveness between the groups.

**Figure 11:**
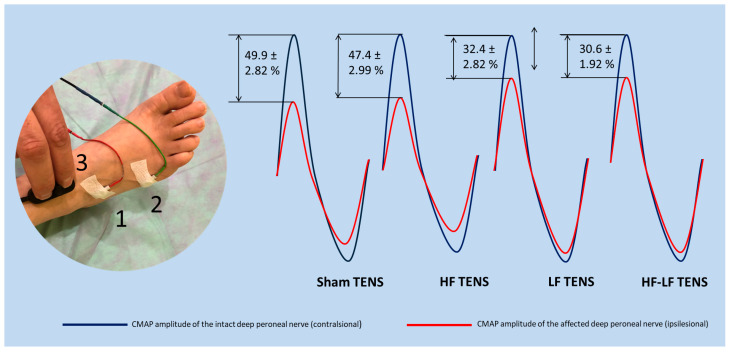
Relative decrease in compound muscle action potential amplitude in response to distal deep peroneal nerve stimulation at the end of the 3 month follow-up. Note: TENS—transcutaneous electrical nerve stimulation; HF—high frequency; LF—low frequency; HF-LF TENS—combination of HF TENS and LF TENS; CMAP—compound muscle action potential.

Comparative analysis between groups revealed that the relative decrease in CMAP amplitude was maximal in the groups that received sham TENS and HF TENS, and it reached an average of 48.6%. Comparatively, the relative reduction in CMAP amplitude was reduced by 35.3% (t = 4.37, *p* ≤ 0.01) after LF TENS application, and it averaged 32.4 ± 1.68% in the LF TENS group and 30.6 ± 1.92% in the HF-LF ENS group. No reliable differences were recorded between the results of the sham TENS and HF TENS groups, or between the LF TENS and HF-LF TENS groups (Figure 11).

## 4. Discussion

Our study explored the recovery effect of direct TENS in the treatment of FT syndrome and monitored this effect for 3 months after the end of treatment. In this study, four different TENS modalities were used, sham TENS, HF TENS, LF TENS, and HF-LF TENS, to identify the most effective TENS and conduct a comparative analysis between them. In parallel, pharmacotherapy was carried out for 2 months in all study groups.

A more pronounced analgesic and recovery effect of 44.5% (t = 4.51, *p* < 0.01) was revealed after the use of active TENS (HF TENS, LF TENS, and HF-LF TENS) compared to sham TENS.

### 4.1. Analgesic Effect of TENS

In the present study, active TENS demonstrated a 2.5-fold reduction in pain and paresthesia at rest compared with sham TENS. Numerous studies have concluded that TENS has a pronounced analgesic effect in the treatment of a number of pathological conditions, such as neuropathic pain [27,37,38], fibromyalgia [39], trigeminal neuralgia [40], postoperative pain [41], chemotherapy-induced peripheral neuropathy [42], phantom pain and stump pain following amputation in adults [43], cancer pain [44], low back pain [45], meralgia paresthetica [46], and migraine headaches [47]. However, clinical studies devoted to the treatment of tunnel syndromes by TENS are scarce.

Most studies have focused on carpal tunnel syndrome [26,48,49]. However, no strong body of research has investigated the effect of TENS in the treatment of FT pain syndrome. The analgesic effect of TENS was first substantiated long before the start of the active experimental study of TENS and its mechanisms of action in 1967 [50,51,52]. Noticeably, the repetitive and prolonged discontinuation of central pain input by TENS leads to a dwindling in the excitability of interneurons that form central sensitization [53,54,55,56,57,58]. The correct is: In consequence, for weeks and even months after the end of treatment there is a gradual subsequent reduction in pain syndrome to the level of afferentation from damaged tissue [59]. Moreover, after the application of TENS a decrease in the activity of dorsal horn neurons was observed in healthy animals, as was a reduction in hyperalgesia in animals with acute inflammation [54].

Furthermore, in addition to the segmental effect, TENS has revealed other molecular mechanisms that activate a complex neural network in the periaqueductal gray matter, rostral ventromedial medulla, and spinal cord [53], contributing to a high temporary and prolonged analgesic effect. Of these molecular mechanisms, the release and accumulation of β-endorphins and methionine enkephalin in the cerebrospinal fluid [60], the release of GABA [61], as well as the inhibition of excitatory neurotransmitters glutamate and substance P in the deep dorsal horn of the spinal cord in animals with inflammation [62] have been experimentally demonstrated in many studies.

### 4.2. The Recovery Effect of TENS

As a result of our study, clinical and neurophysiological signs of sensorimotor recovery of the CPN were identified only after active TENS, and not after sham TENS. Compelling evidence has been obtained for the great recovery potential of direct TENS in the treatment of other compression neuropathies, particularly in the treatment of carpal tunnel syndrome [26,49,63,64,65], meralgia paresthetica [46,66], Bell’s palsy [67], and cubital tunnel syndrome [68]. It is noteworthy that there are few published works devoted to this topic, especially on the treatment of compression neuropathies of the lower extremities.

Our results are in line with clinical reports from other authors which indicated not just a regression of clinical sensory and motor disorders but also an improvement in neurophysiological characteristics of affected nerves [66,68].

A special feature of our work is the use of direct TENS in the treatment of patients with FT syndrome in the acute period, when atrophic changes in the affected CPN have not yet had time to form. In this regard, the relative decrease in CMAP amplitude at the end of the follow-up was assessed using evoked ENMG. The results showed that the development of secondary distal axonopathy of the CPN in the effective TENS group was significantly lower, 31.5% lower, compared with the sham TENS group. These findings are in conformity with previously proven facts about the limited spread of the denervation process, with the maintenance of a greater number of functioning motor units after effective TENS working in parallel with the reinnervation of the denervated muscle through collateral sprouting of adjacent surviving motor units or through the resumption of axon growth from the site of injury [24,68,69].

### 4.3. Comparative Analysis Between HF TENS and LF TENS

The analgesic effect was higher after HF TENS compared to LF TENS, but this effect was not sustainable and gradually regressed after the procedures were stopped. Interestingly, the analgesic effect of LF-TENS continued to increase even after the end of the treatment course. The uniqueness of each modality is associated with the distinctive action characteristics of each of them on the neurobiological mechanisms of pain and analgesia. The analgesic effect of HF TENS is segmental in nature. It cannot be concluded with certainty that LF-TENS does not have a segmental analgesic effect since all impulses pass through the spinal cord. However, a comparative analysis of stimulation frequencies and the resulting analgesic effect showed that reducing the stimulation frequency from 50 Hz to 1 Hz does not reduce the analgesic effect by 50 times but instead reduces it by only 23%. This equation certainly suggests the presence of other analgesic mechanisms in LF TENS besides segmental ones. Extrasegmental analgesic mechanisms of LF TENS are related to the release of beta endorphin in the central nervous system [70]. Recently, researchers have suggested that the prefrontal cortex and limbic system respond differently to pain depending on emotional states. Most often, this may lead to an increase in the intensity of pain perception regardless of the strength and frequency of nociceptive afferentation [71,72,73]. Currently, the anxiolytic [74], anti-affective [49], and anti-mental stress [75] effects of LF TENS have been proven in separate clinical studies, which may explain its additional analgesic effect.

Prominently, the study’s outcomes revealed a by 34.7% greater recovery in sensory function in patients after undergoing HF TENS compared to LF TENS. However, during the 3 months after completion of TENS therapy the stability of the results obtained in the HF TENS group was accompanied by a gradual decrease in sensory impairments in the LF TENS group, which ultimately led to an excess of the recovery effect of 33.4% in the LF TENS group over the HF TENS group. In our opinion, the more pronounced sensory recovery effect of HF-TENS, recorded immediately after treatment, is partly associated with its high analgesic effect. Many studies explain the development of functional hypoesthesia in patients with severe pain to non-noxious mechanical stimuli using the hyposensitivity of the somatosensory system associated with central sensitization [76,77,78,79,80]. However, this hypoesthesia is reversible and regresses after pain subsides [76,81]. Apparently, the stability of this effect can be explained by the persistence of regenerative mechanisms in the structure of sensory fibers even after the end of treatment. A number of authors believe that TENS appears to only speed up axonal regeneration [82]. Notably, the recovery effect was more accelerated after using LF TENS, which gives it more advantages in the long term.

Distinctive features of the motor and gait recovery in our study are due to the reactions of motor fibers, primarily to the effects of LF TENS. These effects are supported by existing research demonstrating LF TENS’s ability to improve nerve regeneration, accelerate reinnervation [83,84], increase angiogenesis [85], and express and accumulate nerve growth factor and brain-derived neurotrophic factor [86] in the area of TENS application. One of the most important results of our research is the results of evoked EMG examination, which proved the ability of LF-TENS to protect damaged nerve fibers from atrophy in the acute period of compression neuropathy. Simultaneously with the protective effect of LF TENS, regenerative changes in damaged nerve and denervated muscle were recorded by needle EMG, which corroborate previous research [66,68]. However, as far as we know, such work has not been carried out previously.

### 4.4. The Benefit of HF TENS and LF TENS Combination

The analgesic effect of HF-LF TENS combines the maximum analgesic effect of HF TENS obtained after treatment and the maximum accumulated analgesic effect of LF TENS by the end of the follow-up. Thus, the maximum analgesic effect is achieved after the completion of the procedures, which continues to increase during 3 months of follow-up. The most important characteristic of HF-LF TENS is the simultaneous recovery effect of sensory and motor disorders, with a maximum effect that is not inferior to the maximum effect due to the separate use of HF TENS or LF TENS. In our opinion, the combination of HF TENS and LF TENS increases the range of maximum therapeutic effects of TENS, which ultimately turned out to be stronger in improving the quality of life of patients compared to separate use of HF and LF TENS by 65.7% after treatment and by 50.4% 3 months after completion of TENS therapy. Our results are in consonance with the findings of other authors [49,87].

## 5. Limitations

This study has several limitations. A blinding assessment was not conducted during the trial study. In addition, to determine the sample size due to the lack of literature on the treatment of FT syndrome with TENS, we used the results of a previous study on the effectiveness of TENS in the treatment of persistent sensory and motor deficits after carpal tunnel release. Another limitation of our study is that sham TENS was applied with minimal imperceptible sensory input which could have slightly affected the analgesic system, but it did not raise doubts among patients about the effectiveness of the treatment used. The second important and distinctive feature of sham TENS is the fixation of electrodes, which was away from the area of the pathological process and was localized on the projection of the anterior surface of the tibial muscles and ankle joint, and not above the CPN in the FT. Another potential limitation of this study is the use of data transformations, normalization, and outlier limitation in statistical analyses of secondary outcome measures to adjust for different baseline values between groups. Moreover, the results of EMG examination are considered as exploratory endpoints. Therefore, the results of secondary and exploratory endpoints require further study in this direction.

## 6. Conclusions

Early use of TENS in the treatment of FT syndrome within 2 weeks after onset turned out to be highly effective compared to sham TENS in reducing hypoesthesia, motor deficit, and gait disturbance. The analgesic effect and sensory recovery were higher after HF TENS. Motor and gait disturbances were reduced only after LF TENS, with evidence of prolonged regenerative and protective effect for at least 3 months after the end of treatment. The combination of HF TENS and LF TENS increases the therapeutic range of TENS, with the achievement of the maximum positive effect of HF TENS and LF TENS after treatment and during the long-term period which subsequently leads to a more pronounced improvement in the quality of life of patients with this pathology.

## Figures and Tables

**Table 1:** Demographic and clinical aspects of the study groups.

	Sham TENS	HF TENS	LF TENS	HF-LF TENS	*p*
Number of patients in each group	20	20	21	21	
Age (years)	39.7 ± 2.88	41.2 ± 2.95	42.0 ± 3.14	39.1 ± 2.90	*p* = 0.897
Gender (female: male)	(10:10)	(11:9)	(11:10)	(11:10)	*p* = 0.992
Disease duration (days)	8.05 ± 0.7	8.24 ± 0.6	8.15 ± 0.7	8.10 ± 0.7	*p* = 0.998
Ankle dorsiflexion weakness (scores)	3.20 ± 0.17	3.10 ± 0.18	3.15 ± 0.18	3.10 ± 0.19	*p* = 0.975
Steppage gait disorder (points)	2.90 ± 0.06	2.86 ± 0.07	2.87 ± 0.07	2.87 ± 0.07	*p* = 0.978
Hypoesthesia (points)	6.10 ± 0.28	6.31 ± 0.29	6.25 ± 0.31	6.20 ± 0.27	*p* = 0.963

Note: TENS—transcutaneous electrical nerve stimulation; HF—high frequency; LF—low frequency; HF-LF TENS—combination of HF TENS and LF TENS; *p*—borderline significance, mean ± standard error of the mean.

**Table 2:** Characteristics and techniques of transcutaneous electrical nerve stimulation application in the study groups.

	F	D	A	Stable Stimulation	Labile Stimulation
Sham TENS	I Hz	50 μs	Sensory response	Cathode fixed above the medial level of tibial anterior muscle. Anode fixed above the ankle.	Cathode fixed above the medial level of tibial anterior muscle. Anode was unfixed and moved around the cathode in 2 cm increments, with stimulation at each point lasting 20 s.
HF TENS	50 Hz	50 μs	Sensory response	Anode fixed over the CPN at the proximal entrance of fibular tunnel Cathode fixed above CPN at the distal entrance of fibular tunnel.	Anode fixed over the CPN at the proximal entrance of fibular tunnel Cathode was unfixed and moved along the peroneal nerve from anode distally in 2 cm increments, with stimulation at each point lasting 20 s.
LF TENS	1 Hz	200 μs	Motor response	Cathode fixed over the CPN at the proximal entrance of fibular tunnel Anode fixed above CPN at the distal entrance of fibular tunnel	Cathode fixed over the CPN at the proximal entrance of fibular tunnel Anode was unfixed and moved along the peroneal nerve from anode distally in 2 cm increments, with stimulation at each point lasting 20 s.
HF-LF TENS	A combined application of HF TENS and LF TENS was used				

Note: TENS—transcutaneous electrical nerve stimulation; HF—high frequency; LF—low frequency; HF-LF TENS—combination of HF TENS and LF TENS; F-frequency; D—duration; A—amplitude.

## Data Availability

The original contributions presented in this study are included in the article. Further inquiries can be directed to the corresponding authors.
